# C5a and its receptors in human anti-neutrophil cytoplasmic antibody (ANCA)-associated vasculitis

**DOI:** 10.1186/ar3873

**Published:** 2012-06-12

**Authors:** Jun Yuan, Shen-Ju Gou, Jing Huang, Jian Hao, Min Chen, Ming-Hui Zhao

**Affiliations:** 1 Renal Division, Department of Medicine, Peking University First Hospital, Institute of Nephrology, Peking University, Key Laboratory of Renal Disease, Ministry of Health of China, Beijing 100034, China; 2 Renal Division, Department of Medicine, Hubei Provincial Hospital of Traditional Chinese Medicine, Department of Internal Medicine, Hubei University of Traditional Chinese Medicine, Wuchang 430061, Wuhan City, China

## Abstract

**Introduction:**

The complement system is crucial for the development of antineutrophil cytoplasmic antibody (ANCA)-associated vasculitis (AAV). In particular, C5a plays a central role. In this study, plasma and urinary levels of C5a as well as renal C5a receptors (CD88 and C5L2) expression were investigated in patients with AAV.

**Methods:**

Twenty-four patients with AAV in the active phase, 19 patients with AAV in the remission phase, and 20 patients with lupus nephritis (LN) were included. Plasma and urinary levels of C5a were measured with enzyme-linked immunosorbent assay (ELISA). The staining of CD88 and C5L2 in renal specimens was detected with immunohistochemistry.

**Results:**

The level of plasma C5a was significantly higher in patients with AAV in the active phase than that in patients in remission, that in patients with LN, and that in normal controls. The urinary C5a level was significantly higher in patients with AAV in the active phase than that in patients in remission and that in normal controls, but not significantly different between patients with active AAV and patients with LN. The mean optical density of CD88 staining in the tubulointerstitium was significantly lower in AAV patients than that in normal controls (0.0052 ± 0.0011 versus 0.029 ± 0.0042; *P *= 0.005). The mean optical density of C5L2 in glomeruli was significantly higher in AAV patients than that in normal controls (0.013 ± 0.0027 versus 0.0032 ± 0.0006; *P *< 0.001). The mean optical density of CD88 staining closely correlated with the initial eGFR (*r *= 0.835; *P *< 0.001) in AAV patients. Double-labeling immunofluorescence assay suggested that CD88 did not express on neutrophils, monocytes, or macrophages, but C5L2 expressed on neutrophils (or monocytes) and macrophages.

**Conclusion:**

The elevated plasma and urinary C5a levels indicated complement activation in human AAV. The level of renal CD88 expression could reflect the disease severity of ANCA-associated glomerulonephritis. CD88 expression was downregulated, and C5L2 was upregulated in ANCA-associated glomerulonephritis.

## Introduction

Antineutrophil cytoplasmic antibodies (ANCAs)-associated vasculitis (AAV) comprises a group of autoimmune disorders, including granulomatosis with polyangiitis (GPA, previously named Wegener granulomatosis), microscopic polyangiitis (MPA), Churg-Strauss syndrome (CSS), and renal-limited vasculitis (RLV) [1]. These diseases are characterized by necrotizing small-vessel vasculitis. ANCAs are the serologic hallmarks for the previously mentioned primary small-vessel vasculitis. ANCAs are predominantly immunoglobulin G (IgG) autoantibodies directed against neutrophil cytoplasmic constituents, in particular, proteinase 3 (PR3) and myeloperoxidase (MPO) [1].

The histopathologic hallmark of ANCA-associated glomerulonephritis is "pauci-immune" necrotizing crescentic glomerulonephritis (NCGN), characterized by little or no glomerular staining for immunoglobulins and complements in renal histology by immunofluorescence microscopy examination. Recent studies in a mouse model of anti-MPO IgG-mediated glomerulonephritis suggested that complement activation via the alternative pathway was crucial for the disease development [2,3]. In particular, Schreiber *et al. *[4] further found that recombinant C5a dose-dependently primes neutrophils for an ANCA-induced respiratory burst. In animal models, C5a receptor (C5aR)-deficient animals were protected from ANCA-induced NCGN. As such, the interaction between C5a and C5aR (CD88) may compose an amplification loop and, thus, plays a central role in ANCA-mediated neutrophil recruitment and activation [4]. However, the role of interaction between C5a and its receptors in the pathogenesis of human AAV is less clear.

C5a is a cleavage product of complement C5 with chemotactic and anaphylatoxic properties. C5a exerts its action through two different receptors: C5aR (CD88) and C5a receptor-like 2 (C5L2), each of which can bind C5a with high affinity [5]. CD88 contributes to the initiation of acute inflammatory responses, such as chemotaxis, enzyme release, and the respiratory burst [5,6]. In contrast, C5L2 seems to have antiinflammatory functions by reducing the C5a available binding to CD88, so it is called a "default" or "scavenger" receptor [5,6]. However, the role of C5L2 is much more unclear and is inconsistent in different diseases [7]. It has been reported that C5L2 is implicated in the inflammatory response in ovalbumin-induced asthma [7]. To the best of our knowledge, C5L2 has not been investigated in AAV. In the current study, plasma and urinary levels of C5a as well as renal C5a receptors (CD88 and C5L2) expression were investigated in patients with ANCA-associated pauci-immune NCGN.

## Materials and methods

### Patients

Twenty-four consecutive patients with AAV in the active phase of initial onset before initiation of immunosuppressive therapy and 19 consecutive patients with AAV in the remission phase after immunosuppressive therapy, diagnosed at Peking University First Hospital from 2008 to 2009, were included. All these patients had a positive test for perinuclear ANCA (P-ANCA) by indirect immunofluorescence and MPO-ANCA by antigen-specific enzyme-linked immunosorbent assay (ELISA). All these patients met the Chapel Hill Consensus Conference (CHCC) definition of AAV [8] and had complete clinical data. All the previously mentioned 24 patients with AAV in the active phase received renal biopsy at diagnosis and before immunosuppressive therapy. "Remission" was defined as "absence of disease activity attributable to active disease qualified by the need for ongoing stable maintenance immunosuppressive therapy" (complete remission), or "50% reduction of disease activity score and absence of new manifestations" (partial remission), as described previously [9]. Patients with secondary vasculitis or with any other coexisting renal disease were excluded.

Twenty-five age- and gender-matched healthy blood donors and 20 patients with biopsy-proven lupus nephritis (LN), diagnosed in the same period in our center, were enrolled as normal control and disease control, respectively. All the patients with lupus nephritis fulfilled the 1997 American College of Rheumatology revised criteria for systemic lupus erythematosus (SLE) [10]. Of the 20 patients with lupus nephritis, six were classified as type III, and 14 were classified as type IV, according to the abbreviated version of the ISN/RPS classification [11].

Estimated glomerular filtration rate (eGFR) was calculated by using the modification of diet in renal disease (MDRD) equations [12].

The research was in compliance of the Declaration of Helsinki and approved by the ethics committee of our hospital. Written informed consent was obtained from each participant.

### Plasma and urine samples collection

For patients with AAV in the active phase and patients with lupus nephritis, blood and urinary samples were collected at the day of renal biopsy and before the initiation of immunosuppressive treatment. For patients with AAV in the remission phase, blood samples were collected at their regular ambulatory visits. Peripheral blood was collected by using disodium EDTA as anticoagulant, and plasma was collected after centrifugation. The plasma and urine samples were immediately put on ice after the sample was drawn to prevent complement degradation *ex vivo*. After centrifugation, all the samples were stored in aliquots at -80°C until use. When testing, after rapid thawing at 37°C, the frozen specimens were transferred immediately in ice before use within 1 hour. The specimens were tested without repeated freeze and thaw.

### Isolation of neutrophils

Neutrophils were isolated from heparinized venous blood of healthy donors by density gradient centrifugation on Lymphoprep (Nycomed, Oslo, Norway). Erythrocytes were lysed with ice-cold ammonium chloride buffer, and neutrophils were washed in Hanks balanced salt solution without Ca^2+^/Mg^2+ ^(HBSS^-/-^; Chemical Reagents, Beijing, China). Neutrophils were then suspended in HBSS with Ca^2+^/Mg^2+^(HBSS^+/+^; Chemical Reagents) to a concentration of 2.5 × 10^6 ^cells/ml. Neutrophils were activated by 2 ng/ml tumor necrosis factor (TNF)-α. The specimens were fixed with 70% ethanol for 5 minutes, and permeabilized with 0.5% Triton X-100 for 5 minutes at 4°C.

### Quantification of C5a

Urine and plasma concentrations of human fragment C5a were determined with enzyme-linked immunoassays (Quidel Corporation, San Diego, CA, USA). Optimal dilutions of urine and plasma samples were obtained according to the ELISA kit instructions. The urinary creatinine also was measured. The urinary level of C5a was calculated by the ratio of urinary concentration of C5a to the urinary creatinine.

### Renal histology

The renal histology of patients with AAV in the active phase was evaluated according to the previous standardized protocol [13-15]. The presence of glomerular lesions, including fibrinoid necrosis, crescents, and glomerulosclerosis, was calculated as the percentage of the total number of glomeruli in a biopsy. Interstitial and tubular lesions were scored semiquantitatively on the basis of the percentage of the tubulointerstitial compartment that was affected: interstitial infiltrate ("-"for 0; "+" for 0 to 20%; "++" for 20% to 50%; and "+++" for > 50%), interstitial fibrosis ("-" for 0; "+" for 0 to 50%; and "++"for > 50%) and tubular atrophy ("-" for 0; "+" for 0 to 50%; and "++" for > 50%).

In renal sections stained with hematoxylin/eosin (HE), the numbers of neutrophils were counted in the glomeruli of AAV by an experienced pathologist.

### Renal immunohistochemistry

Five renal tissues were obtained from the normal part of nephrectomized (because of renal carcinoma) kidneys and were used as normal controls. Their renal tissues were considered normal with light microscopy, immunofluorescence, and electron microscopy.

Tissue sections were deparaffinized in xylene and rehydrated through grading alcohols. Slides were immersed in citric acid buffer (0.01 *M*, pH 6.0) and were treated in an 800 W microwave oven for 2 minutes and then at 200 W for 8 minutes, for antigen retrieval. Slides were rinsed in phosphate-buffered saline (0.01% PBS). Endogenous peroxidase was blocked with 0.3% H_2_O_2 _in methanol for 30 minutes at room temperature. After a 30-minute blocking step with 3% normal goat serum diluted in PBS, mouse anti-human CD88 (1:50 dilution; Abcam, Cambridge, UK; catalog number, ab11867), rabbit anti-human C5L2 (1:500 dilution; Abcam, catalog number, ab77982), and mouse anti-human CD66b (1:200 dilution; Abnova, Taiwan; catalog number, MAB7198) were applied and incubated overnight at 4°C in a moisture chamber. After multiple PBS rinses, the slides were exposed to secondary antibodies. The detection system used, Dako EnVision HRP (Dako A/S, Copenhagen, Denmark), was an avidin-free two-step indirect method with goat anti-rabbit and goat anti-mouse immunoglobulins conjugated with horseradish peroxidase (HRP) as secondary antibodies. The secondary antibodies were incubated for 30 minutes at 37°C, and sections were developed in fresh hydrogen peroxide plus 3-3-diaminobenzidine tetrahydrochloride solution for 1 minute. As negative controls, primary antibodies were replaced by normal rabbit IgG or normal mouse IgG. Finally, the sections were incubated with hematoxylin and dehydrated through alcohols and xylene. The sections were examined with light microscopy.

In total, 20 fields of vision per kidney section (three to four sections per kidney) at ×200 were observed blindly as a semiquantitive assessment of immunohistochemical staining. The renal CD88 and C5L2 stainings were evaluated with the Image Pro Plus analysis software 6.0. The positive signals were quantified as the mean optical density (integrated option density/area).

### Colocalization of CD88 as well as C5L2 with elastase and CD68

Elastase is the marker for neutrophils and monocytes [16], and CD68 is the marker for macrophages [17]. Colocalization of CD88 and C5L2 with elastase and CD68 was judged by double-labeling immunofluorescence in formalin-fixed paraffin-embedded tissue. The deparaffinage and antigen-retrieval step were consistent with the previous immunohistochemistry described earlier. After a 30-minute blocking step with 3% normal goat serum diluted in PBS, mouse anti-human CD88 (1:5 dilution; Abcam) combined with rabbit anti-human neutrophil elastase (1:50 dilution; Abcam) or with rabbit anti-human CD68 (1:50 dilution; Abbiotec, San Diego, CA, USA), and rabbit anti-human C5L2 (1:50 dilution; Abcam) combined with mouse anti-human neutrophil elastase (1:5 dilution; Genway Biotech, San Diego, CA, USA) or with mouse anti-human CD68 (1:5 dilution; Abcam) were added and incubated overnight at 4°C in a moisture chamber, 3 × 5-minute washes in PBS. The secondary antibodies FITC-labeled goat anti-mouse IgG and TRITC-labeled goat anti-rabbit IgG (both 1:60; Southern Biotech) were incubated for 30 minutes at 37°C in a moisture chamber, 3 × 5-minute washes in PBS. Nuclei were stained with DAPI (ZSGB, Beijing, China) and sections mounted with Citifluor. In negative controls, primary antibodies were replaced by normal rabbit IgG or normal mouse IgG.

The spleen and neutrophils were used as positive controls. Neutrophils were isolated from peripheral blood of healthy blood donors and fixed with 70% ethanol. The spleen tissues were obtained from a patient subjected to splenectomy for traumatic splenic rupture. The spleen tissues were fixed in 10% buffered formalin, processed through graded alcohols and xylene, and embedded in paraffin. For the primary antibody, mouse anti-human CD88 (1:20 dilution for neutrophil-coated slides and 1:5 dilution for spleen tissues; Abcam) combined with rabbit anti-human neutrophil elastase (1:50 dilution for neutrophils and spleen; Abcam) were used. The other steps were consistent with aforementioned method.

The stained sections were visualized with fluorescence microscopy (80i; Nikon, Tokyo, Japan). Images were exported from the Nikon fluorescence microscopy software.

### Hematoxylin/eosin and CD88 counterstaining

To confirm whether CD88 was expressed on renal infiltrated neutrophils of AAV patients, the renal sections of AAV were counterstained with HE on the basis of immmunohistochemical staining of CD88 (1:50 dilution; Abcam; catalog number, ab11867). The sections were examined with light microscopy.

### Statistical analysis

Differences of quantitative parameters between groups were assessed by using the *t *test (for normally distributed data) or nonparametric test (for non-normally distributed data). Spearman correlation was used to measure the relation between two non-normally distributed variables or between one non-normally distributed variable and one normally distributed variable. Pearson correlation was used to measure the relation between two normally distributed variables. The difference was considered significant if the *P *value was < 0.05. Analysis was performed with the SPSS statistical software package (version 13; Chicago, IL, USA).

## Results

### Demographic and clinical data

Among the 24 patients with AAV in the acute phase, 11 were male and 13 were female patients, with an age of 55.4 ± 15.7 years (range, 14 to 82 years) at diagnosis. Of the 24 patients, the level of initial serum creatinine was 297.0 ± 232.8 (range, 59 to 1,007) μ*M*; the level of urinary protein excretion was 2.56 ± 1.72 g/24 hours (range, 0.23 to 7.35 g/24 hours). Twenty patients had renal insufficiency at diagnosis. The organ involvements are listed in Table 1.

**Table 1:** Clinical data of patients with ANCA-associated vasculitis

Parameters	Number
Male/female	11/13
Average age at onset of the disease (years)	55.364 ± 15.72
Serum creatinine (SCr; μ*M*)	
Mean ± SD	296.99 ± 232.83
Range	59 to 1,007
eGFR (ml/min/1.73 m^2^)	
Mean ± SD	31.98 ± 31.48
Range	0.7 to 118
Renal insufficiency at diagnosis	20 (83.3%)
Urinary protein (g/24 hours)	
Mean ± SD	2.56 ± 1.72
Range	0.23 to 7.35
Skin rash	2 (8.3%)
Arthralgia	8 (33.3%)
Muscle pain	6 (25.0%)
Pulmonary	10 (41.2%)
ENT	12 (50.0%)
Ophthalmic	5 (20.8%)
Gastrointestinal	4 (16.7%)
Nervous system	2 (8.3%)
BVAS	20.46 ± 4.93

BVAS, Birmingham Vasculitis Activity Score; eGFR, estimated glomerular filtration rate; ENT, ear, nose, and throat; SD, standard deviation.

Among the 19 patients with AAV in the remission phase, nine were male and 10 were female patients, with an age of 62.1 ± 13.9 years (range, 30 to 81 years) at diagnosis. The level of serum creatinine was 165.3 ± 99.3 (range, 58 to 441) μ*M*. The level of Birmingham Vasculitis Activity Scores [18] (BVAS) was 0 and 2 in 18 and one patients, respectively.

### Renal histopathology

Among the renal biopsies of the 24 patients with the active phase of AAV, all had little or no staining for IgG, IgA, and IgM by immunofluorescence microscopy (≤ 1+ on a scale of 0 to 4+). No or little electron-dense deposit was detected with electron microscopy. The glomerular and tubulointerstitial lesions are listed in Table 2.

**Table 2:** Renal histopathology of patients with ANCA-associated vasculitis

Glomerular lesions	Percentage
Normal glomeruli	37.90 ± 37.40
Crescents	62.09 ± 31.69
Cellular crescents	16.39 ± 16.52
Fibrocellular crescents	33.66 ± 24.43
Fibrous crescents	12.04 ± 20.00
Glomerular sclerosis (median and range)	5.26 (0 to 30.0)

**Tubulointerstitial lesions**	**Number**
Interstitial infiltration (-/+/++/+++)	1/4/4/15
Interstitial fibrosis (-/+/++)	1/8/15
Tubular atrophy (-/+/++)	1/7/16

We found 2.56 ± 0.14 neutrophils per glomeruli of AAV in HE staining sections, whereas no neutrophils were present in glomeruli of normal controls (see Additional file 1, Figure S1).

### Plasma and urinary C5a levels

Plasma samples were obtained from 24 patients with AAV in the active phase, 19 patients with AAV in the remission phase, 20 patients with LN, and 25 healthy blood donors. Urinary levels of C5a were measured in 24 patients with AAV in the active phase, 19 patients with AAV in remission, 10 patients with LN, and 25 healthy blood donors.

**Figure 1:**
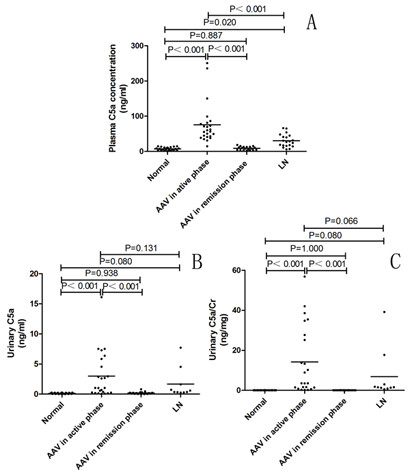
**Plasma and urinary levels of C5a**. The horizontal lines in the graph represent the mean values. **(A) **Plasma level of C5a. **(B) **Urinary level of C5a. **(C) **Urinary level of C5a adjusted for urinary creatinine. AAV, ANCA-associated vasculitis; LN, lupus nephritis.

The level of plasma C5a was significantly higher in patients with AAV in the active phase than that in patients with AAV in remission, patients with LN, and normal controls (75.67 ± 11.93 ng/ml versus 8.78 ± 1.12 ng/ml; *P *< 0.001; 75.67 ± 11.93 ng/ml versus 30.42 ± 4.07 ng/ml; *P *< 0.001; 75.67 ± 11.93 ng/ml versus 7.37 ± 0.90 ng/ml; *P *< 0.001, respectively). No significant differences in C5a levels were found between normal control and patients with AAV in remission (8.78 ± 1.12 ng/ml versus 7.37 ± 0.90 ng/ml; *P *= 0.887) (Figure 1).

The urinary C5a level was significantly higher in patients with AAV in the active phase than that in patients with AAV in remission and normal controls (3.00 ± 0.78 ng/ml versus 0.18 ± 0.044 ng/ml, *P *< 0.001; 3.00 ± 0.78 ng/ml versus 0.13 ± 0.02 ng/ml, *P *< 0.001, respectively). This was also the case after adjustment for urinary creatinine (14.13 ± 3.54 ng/mg Cr versus 0.023 ± 0.0058 ng/mg Cr; *P *< 0.001; 14.13 ± 3.54 ng/mg Cr versus 0.0246 ± 0.0037 ng/mg Cr; *P *< 0.001, respectively). No significant difference was noted in urinary C5a levels between patients with AAV in the active phase and patients with LN (Figure 1). No significant correlation was found between the levels of plasma C5a and urinary C5a in patients with AAV.

### Immunohistochemistry for CD88, C5L2, and CD66b

**Figure 2:**
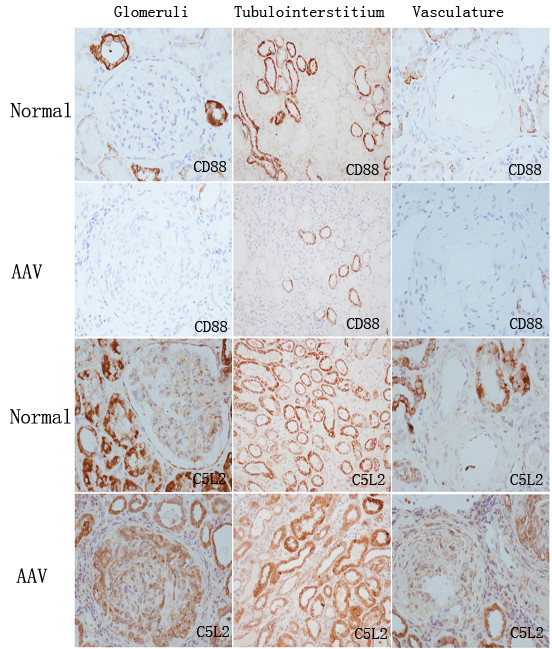
**Immunohistochemistry for CD88 and C5L2 in renal specimens**. (Glomeruli, magnification ×400; tubulointerstitium, magnification ×200; vasculature, magnification ×400).

Studies on renal histopathology were performed in patients with AAV in the active phase. In renal specimens of patients with AAV, immunohistochemical examination revealed prominent expression of CD88 in proximal tubules, distal tubules, and collecting ducts. Expression of CD88 protein was scanty in glomeruli and vasculature of the normal controls and in the patients with AAV (Figure 2).

In contrast, expression of C5L2 was detected in the glomeruli and vasculature. The positive staining of C5L2 was also seen in proximal tubules, distal tubules, collecting ducts, and interstitium (Figure 2).

Immunohistochemical examination showed the prominent expression of CD66b, which is an ideal marker for neutrophils, in glomeruli of AAV patients. Expression of CD66b was scanty in the glomeruli of normal controls (see Additional file 2, Figure S2). It indicated that a number of neutrophils were present in the glomeruli of AAV patients, but no or few neutrophils in the glomeruli of normal controls.

CD88 and C5L2 staining in renal specimens were evaluated by the Image Pro Plus analysis software (version 6.0; Dallas, TX, USA). The mean optical density of CD88 in patients with AAV in the tubulointerstitium was significantly lower than that in normal controls (0.0052 ± 0.0011 versus 0.029 ± 0.0042; *P *= 0.005). The mean optical density of C5L2 in glomeruli was significantly higher in patients with AAV than that in normal controls (0.013 ± 0.0027 versus 0.0032 ± 0.0006; *P *< 0.001). However, no significant difference was seen for the mean optical density of C5L2 in the tubulointerstitium between patients with AAV and normal controls (0.034 ± 0.005 versus 0.044 ± 0.006; *P *= 0.227).

Among the patients with AAV, correlation analysis showed that the mean optical density of CD88 correlated with initial eGFR (*r *= 0.835; *P *< 0.001), and inversely correlated with initial serum creatinine (*r *= -0.628; *P *< 0.001). The mean optical density of CD88 in patients with interstitial infiltrate greater than 50% was significantly lower than that in those with interstitial infiltrate ≤ 50% (0.0035 ± 0.0008 versus 0.0070 ± 0.0023; *P *= 0.034).

The mean density of glomerular C5L2 staining correlated with the ratio of glomerular cell number to glomerular area (*r *= 0.561; *P *= 0.004).

### Immunofluorescence double-labeling

**Figure 3:**
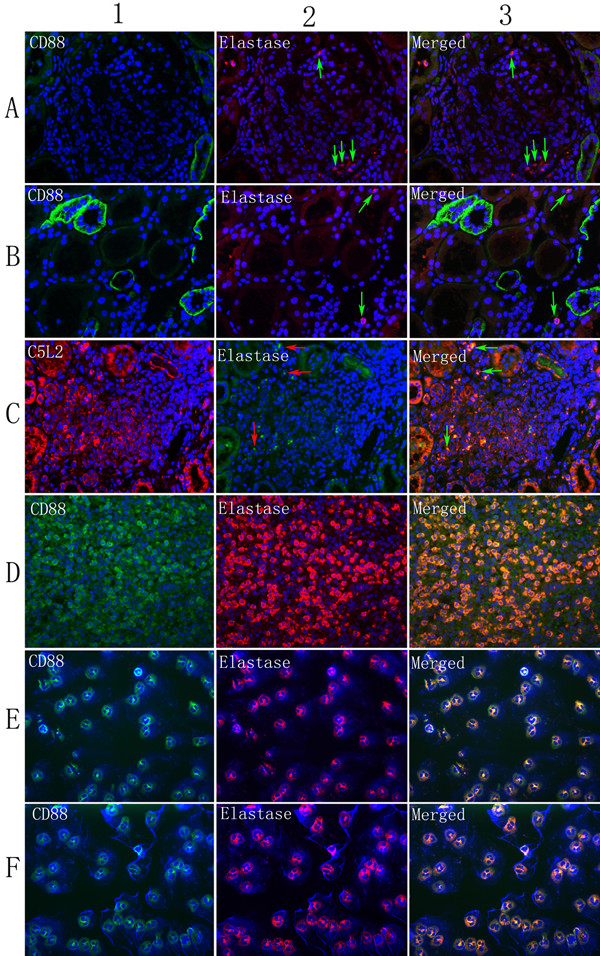
**Colocalization of C5a receptors (CD88 and C5L2) and elastase**. **(A) **Colocalization of CD88 and elastase in glomeruli (arrowheads). **(B) **Colocalization of CD88 and elastase in tubulointerstitium (arrowheads). **(C) **Colocalization of C5L2 and elastase in tubulointerstitium (arrowheads). **(D) **Colocalization of CD88 and elastase in the spleen. **(E) **Colocalization of CD88 and elastase in resting neutrophils. **(F) **Colocalization of CD88 and elastase in activated neutrophils. Magnification ×400.

**Figure 4:**
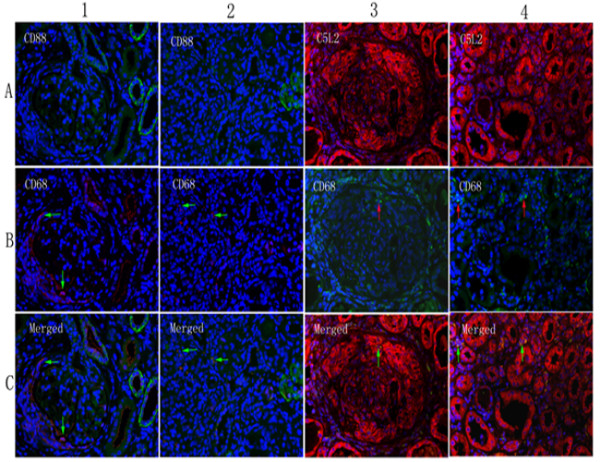
**Colocalization of C5a receptors (CD88 and C5L2) and macrophages**. **(A) **Colocalization of CD88 and CD68 in glomeruli (arrowheads). **(B) **Colocalization of CD88 and CD68 in tubulointerstitium (arrowheads). **(C) **Colocalization of C5L2 and CD68 in glomeruli (arrowheads). **(D) **Colocalization of C5L2 and CD68 in tubulointerstitium (arrowheads). Magnification ×400.

By using immunofluorescence double-labeling with neutrophil elastase or CD68, we determined whether CD88 and C5L2 was expressed on neutrophils (or monocytes) or macrophages. It was shown that little or no CD88 was expressed on neutrophils, monocytes, or macrophages in renal specimens of patients with AAV, and this result was proved by two other anti-CD88 monoclonal antibodies (Santa Cruz Biotechnology, catalog number sc-70812, and Abcam, catalog number ab11884, (see Additional files 3, 4, 5, and 6, Figures S3, S4, S5, and S6). However, CD88 could colocalize with elastase in the spleen- and neutrophil-coated slides. C5L2 expression partly colocalized with neutrophils (or monocytes) and macrophages (Figures 3 and 4).

### HE and CD88 counterstaining

On the basis of immmunohistochemical staining of CD88, the renal sections of AAV were counterstained with HE. Little or no CD88 staining was noted in renal infiltrated neutrophils (see Additional file 7, Figure S7). This was consistent with the results of immunofluorescence double-labeling of CD88 and elastase.

## Discussion

It was previously assumed that the complement system was not involved in the pathogenesis of AAV, because little immunoglobulin or complement deposition is found in AAV, and AAV is generally not associated with hypocomplementemia [19,20]. However, increasing evidence suggests that activation of the complement system, via the alternative pathway, is crucial for the development of AAV [2-4,15,21-23]. The complement system comprises more than 30 plasma and membrane-bound proteins. Among these proteins, C5a, together with its receptor, seems to play a central role in the pathogenesis of AAV, demonstrated by both animal and *in vitro *studies [4]. The current study further investigated C5a as well as its receptor, CD88 and C5L2, in human AAV.

In the current study, only patients with positive MPO-ANCA were included, for two reasons. First, previous studies that investigated the role of the complement system in the pathogenesis of AAV focused mainly on MPO-ANCA-positive vasculitis [2-4,15]. Second, our previous studies suggested that MPO is the most important ANCA target antigen for Chinese patients with AAV; MPO-ANCA-positive patients constituted about 80% to 90% of Chinese patients with AAV [20,24-26].

In the current study, we investigated the plasma and urinary levels of C5a. The potent anaphylatoxin, C5a, is released when C5 is cleaved by C5 convertase [27]. The measurement of cleavage products such as C5a in plasma, therefore, can provide a sensitive index of complement activation. It was found that the plasma level of C5a was about 10 times higher in patients with AAV in the active phase than that in normal controls. In the remission phase of AAV, the plasma level of C5a was comparable to that of normal controls. It might indicate an especially important role of C5a in AAV, compared with some other autoimmune diseases, such as primary antiphospholipid syndrome, in which plasma level of C5a is normal [22]. The urinary level of C5a, after adjustment for urinary creatinine, was even higher (about 560 times) in patients with AAV than that in normal controls. Our results confirmed and further extended a recent observation [28]. Increased urinary C5a might come from two sources. First, C5a may derive from the circulation because of glomerular basement membrane damage. Second, the complement may be activated locally in kidneys, and C5a is therefore released to urine [29,30]. Overall, it may indicate the pathogenic role of excessive or uncontrolled production of C5a in AAV, in particular, in the glomerulonephritis of AAV.

The two types of C5a receptors, CD88 and C5L2, both have high affinity for C5a [6]. Although *in vitro *studies have found that mesangial cells could express CD88 [31], the current study demonstrated that CD88 is expressed mainly in proximal tubules, distal tubules, and collecting ducts, but scarcely at all expressed in glomeruli and vasculature in patients with AAV. Our results are in line with those of previous studies [32-34].

The expression of CD88 in the patients with AAV was significantly lower than that in normal controls, and, more important, was closely correlated with renal function and the extent of interstitial infiltration. Moreover, double-labeling immunofluorescence demonstrated that CD88 is scanty on neutrophils, monocytes, and macrophages. In a different opinion, CD88 is expressed in myeloid cells and is unable to be detected in tubular epithelial cells [35]. However, in the current study, CD88 staining was confirmed by two other anti-CD88 monoclonal antibodies, and similar results were obtained (see Additional files 3, 4, 5, 6, and Figures S3, S4, S5, S6). Moreover, in positive controls, neutrophils could be stained by anti-CD88 monoclonal antibodies. Therefore, our results on neutrophil CD88 staining are reliable.

To confirm that the small amount of glomerular CD88 in AAV patients was not because of the lack of neutrophils in the glomeruli, neutrophils in the glomeruli of AAV patients were confirmed by both HE staining and immunohistochemical staining of CD66b (see Additional file 1, Figure S1; Additional file 2, Figure S2). Obviously, more neutrophil infiltration was found in the renal section of AAV patients than in normal controls. Immunohistochemical examination showed prominent expression of CD66b in glomeruli of AAV patients, whereas expression of CD66b was scanty in glomeruli of normal controls. Moreover, in HE and CD88 counterstaining, little or no CD88 staining was noted in renal infiltrated neutrophils (see Additional file 7, Figure S7). Therefore, it was clear that the small amount of CD88 in glomeruli of AAV patients was because neutrophils in the glomeruli "lost" CD88, not because of the lack of neutrophils in the glomeruli.

Schreiber *et al. *[4] suggested that CD88 in cells of myeloid origin plays a crucial role in the development of AAV. It is well known that CD88 on neutrophils interacts with C5a to produce a series of functional responses such as chemotaxis, enzyme release, degranulation, and respiratory burst [6]. All of these functions induce inflammatory responses and are involved in ANCA-mediated tissue damage [4]. However, the precise role of CD88 in renal tubular epithelial cells in AAV patients remains less defined. In the renal ischemia-reperfusion injury model, the interaction between C5a and CD88 on tubular epithelial cells induces a local inflammatory response resulting in cellular dysfunction and leads to renal function loss [36]. CD88 is known to be rapidly internalized after treatment with C5a [37]. The low expression of CD88 in AAV patients might be attributed to C5a-mediated internalization. The downregulation of CD88 expression in AAV patients might contribute to the self-protection mechanism, so as to alleviate the C5a-mediated inflammation. We speculate that binding of C5a to the CD88 in renal tubular epithelial cells may also be involved in tubulointerstitial injury in AAV patients. In AAV, the role of CD88 expression in the tubulointerstitium and the mediators responsible for downregulation of CD88 expression in the tubulointerstitium await further studies.

In contrast, the role of C5L2 remains controversial. C5L2 has been shown to be a nonsignaling decoy receptor and to compete with CD88 for binding to C5a. Thereby, C5L2 attenuates the proinflammatory C5a response. However, a study demonstrated that C5L2 is involved in the pathogenesis of asthma-like airway hyperresponsiveness and inflammation [7]. Thus, C5L2 plays complex and dual roles in the pathogenesis of inflammation. To the best of our knowledge, the current study was the first one to investigate C5L2 in AAV. We found that the expression of C5L2 in glomeruli in AAV patients is significantly higher than that in normal controls. In ANCA-associated glomerulonephritis, glomerular intrinsic cells proliferation was not common. To some extent, the increase of cells in the glomerular tuft can reflect the glomerular inflammatory infiltrate. In our study, the expression of C5L2 in glomeruli positively correlated with the numbers of cells in the glomeruli. Moreover, C5L2 also expressed on neutrophils (or monocytes) and macrophages, besides on renal intrinsic cells. The significantly increased expression of C5L2 in renal specimens suggested that expression of C5L2 is regulated differently from that of CD88 in AAV. Unlike CD88, C5L2 did not show internalization on C5a binding [38]. Moreover, inflammatory cells (that is, neutrophils, monocytes, and macrophages) infiltrated in kidneys could enhance the C5L2 expression. Whether C5L2 participated in proinflammatory or antiinflammatory responses in AAV requires further study.

## Conclusions

In conclusion, the elevated plasma and urinary C5a levels indicated complement activation in human AAV. The level of renal CD88 expression could reflect the disease severity of ANCA-associated glomerulonephritis. CD88 expression was downregulated, and C5L2 was upregulated in ANCA-associated glomerulonephritis. The exact mechanism of the regulation of CD88 and C5L2 in AAV requires further study.

## Abbreviations

AAV: ANCA-associated vasculitis; BVAS: Birmingham Vasculitis Activity Scores; C5aR: C5a receptor; C5L2: C5a receptor-like 2; CSS: Churg-Strauss syndrome; eGFR: estimated glomerular filtration rate; GPA: granulomatosis with polyangiitis; LN: lupus nephritis; MPA: microscopic polyangiitis; MPO: myeloperoxidase; NCGN: necrotizing crescentic glomerulonephritis; PR3: proteinase 3; RLV: renal-limited vasculitis; SLE: systemic lupus erythematosus.

## Competing interests

The authors declare that they have no competing interests.

## Authors' contributions

MC was involved in all the aspects of study conception, design, and direction, and provided final approval of the version of the submitted manuscript. MHZ was involved in the direction of the study. JY, JH, SJG, and JH performed the experiments, and JY was involved in data acquisition, analysis, and report drafting, and provided the submitted manuscript. All authors read and approved the manuscript.

## Supplementary Material

Additional file 1 **Figure S1**. HE staining in renal sections of AAV Neutrophils, arrowheads. Magnification, ×400. Click here for file

Additional file 2 **Figure S2**. Immmunohistochemical staining of CD66b. **(A) **Immmunohistochemical staining of CD66b in renal sections of AAV. Arrowheads, CD66b-positive cells. **(B) **Immmunohistochemical staining of CD66b in renal sections of normal controls. **(C) **Immmunohistochemical staining of CD66b in spleen. Magnification, ×400. Click here for file

Additional file 3 **Figure S3**. Colocalization of CD88 and elastase in renal specimens. **(A) **Colocalization of CD88 and elastase by Abcam antibody (catalog number, ab11884) (arrowheads). **(B) **Colocalization of CD88 and elastase by Santa Cruz antibody (catalog number, sc-70812) (arrowheads). Magnification, ×400. Click here for file

Additional file 4 **Figure S4**. Colocalization of CD88 and elastase in the spleen. **(A) **Colocalization of CD88 stained by Abcam antibody (catalog number, ab11884) and elastase in the spleen **(B) **Colocalization of CD88 stained by Santa Cruz antibody (catalog number, sc-70812) and elastase in the spleen. Magnification, ×400. Click here for file

Additional file 5 **Figure S5**. Co-localization of CD88 and elastase in neutrophil-coated slides. **(A) **Colocalization of CD88 and elastase in resting neutrophils by Abcam antibody (catalog number, ab11884). **(B) **Colocalization of CD88 and elastase in activated neutrophils by Abcam antibody (catalog number, ab11884). **(C) **Colocalization of CD88 and elastase in resting neutrophils by Santa Cruz antibody (catalog number, sc-70812). **(D) **Colocalization of CD88 and elastase in activated neutrophils by Santa Cruz antibody (catalog number, sc-70812). Magnification, ×400. Click here for file

Additional file 6 **Figure S6**. Immmunohistochemical staining of CD88 in spleen. **(A) **Immmunohistochemical staining of CD88 in spleen by Abcam antibody (catalog number, ab11867). **(B) **Immmunohistochemical staining of CD88 in spleen by Abcam antibody (catalog number, ab11884). **(C) **Immmunohistochemical staining of CD88 in spleen by Santa Cruz antibody (catalog number, sc-70812). Click here for file

Additional file 7 **Figure S7**. Hematoxylin/eosin (HE) and CD88 counterstaining. CD88, open arrow; neutrophils, solid arrow. Magnification, ×400. Click here for file
