# Supplementary figures and images for: C5a and its receptors in human anti-neutrophil cytoplasmic antibody (ANCA)-associated vasculitis
Source: Arthritis Res Ther. 2012 Jun 12;14(3):R140. doi: 10.1186/ar3873 (PMC3446523; doi:10.1186/ar3873)

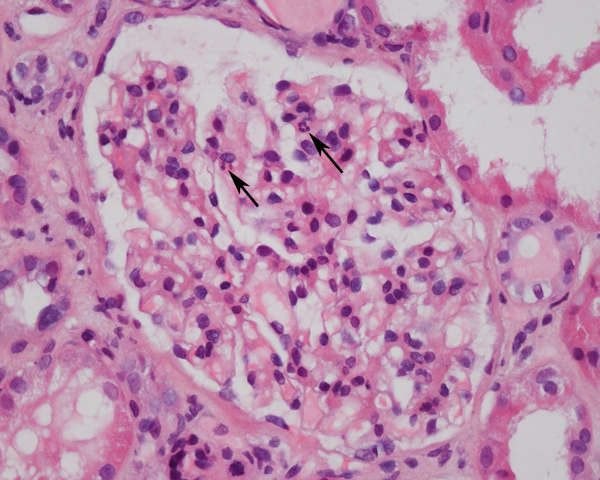

Supplement: Additional file 1 — Figure S1. HE staining in renal sections of AAV Neutrophils, arrowheads. Magnification, ×400. [file ar3873-S1.TIFF]

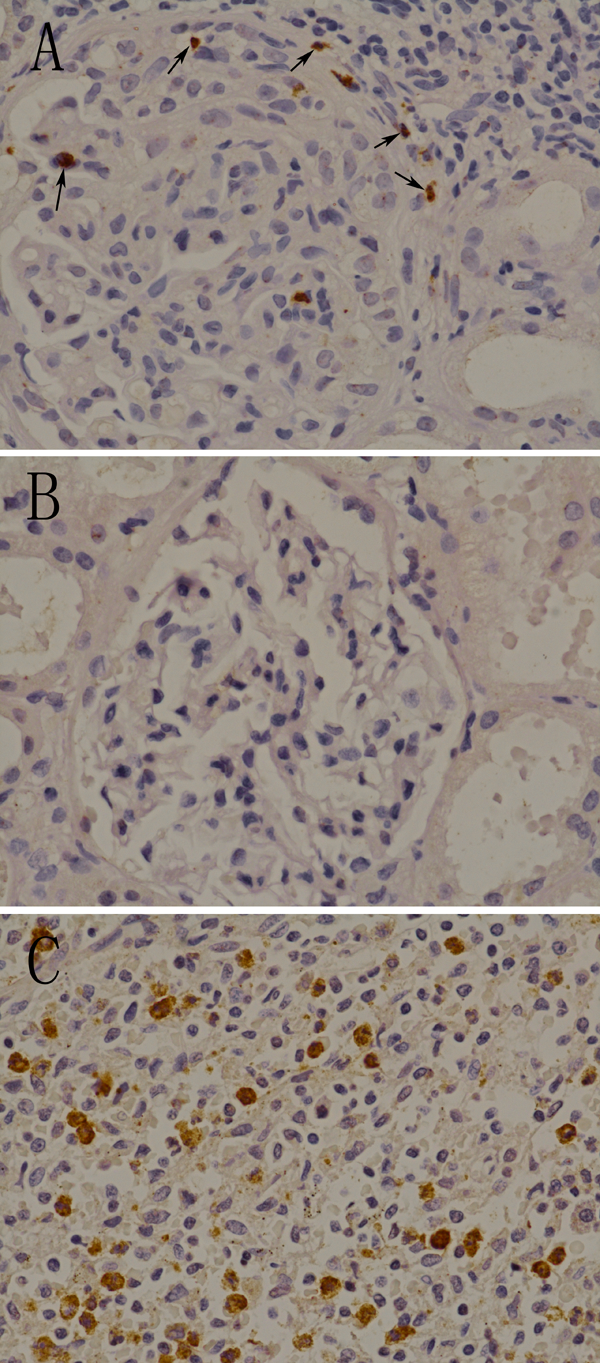

Supplement: Additional file 2 — Figure S2. Immmunohistochemical staining of CD66b. (A) Immmunohistochemical staining of CD66b in renal sections of AAV. Arrowheads, CD66b-positive cells. (B) Immmunohistochemical staining of CD66b in renal sections of normal controls. (C) Immmunohistochemical staining of CD66b in spleen. Magnification, ×400. [file ar3873-S2.TIFF]

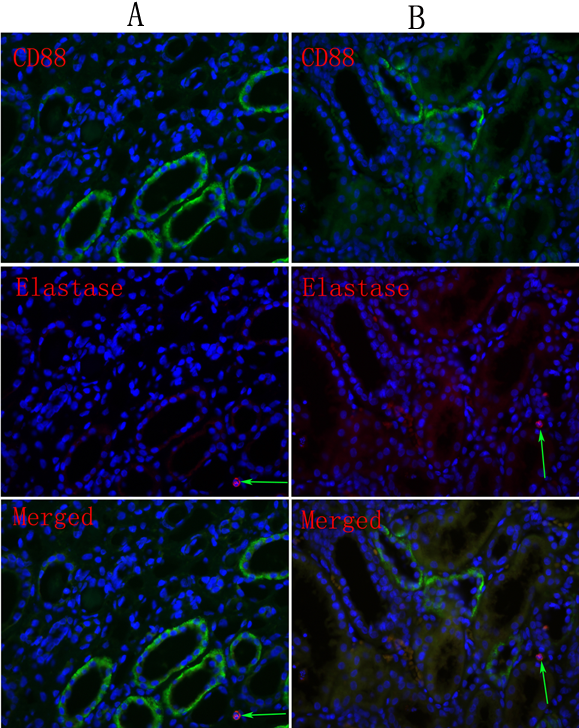

Supplement: Additional file 3 — Figure S3. Colocalization of CD88 and elastase in renal specimens. (A) Colocalization of CD88 and elastase by Abcam antibody (catalog number, ab11884) (arrowheads). (B) Colocalization of CD88 and elastase by Santa Cruz antibody (catalog number, sc-70812) (arrowheads). Magnification, ×400. [file ar3873-S3.TIFF]

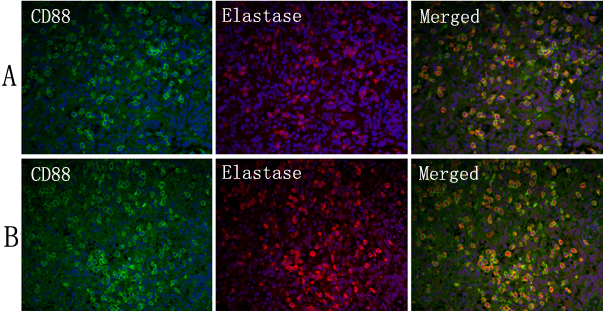

Supplement: Additional file 4 — Figure S4. Colocalization of CD88 and elastase in the spleen. (A) Colocalization of CD88 stained by Abcam antibody (catalog number, ab11884) and elastase in the spleen (B) Colocalization of CD88 stained by Santa Cruz antibody (catalog number, sc-70812) and elastase in the spleen. Magnification, ×400. [file ar3873-S4.TIFF]

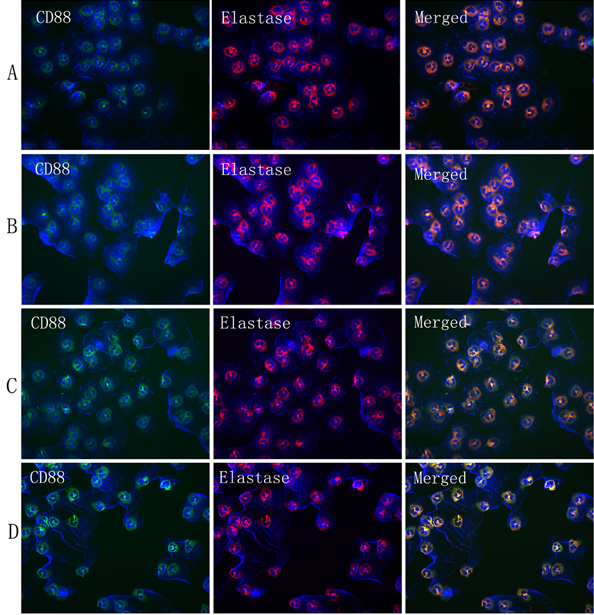

Supplement: Additional file 5 — Figure S5. Co-localization of CD88 and elastase in neutrophil-coated slides. (A) Colocalization of CD88 and elastase in resting neutrophils by Abcam antibody (catalog number, ab11884). (B) Colocalization of CD88 and elastase in activated neutrophils by Abcam antibody (catalog number, ab11884). (C) Colocalization of CD88 and elastase in resting neutrophils by Santa Cruz antibody (catalog number, sc-70812). (D) Colocalization of CD88 and elastase in activated neutrophils by Santa Cruz antibody (catalog number, sc-70812). Magnification, ×400. [file ar3873-S5.TIFF]

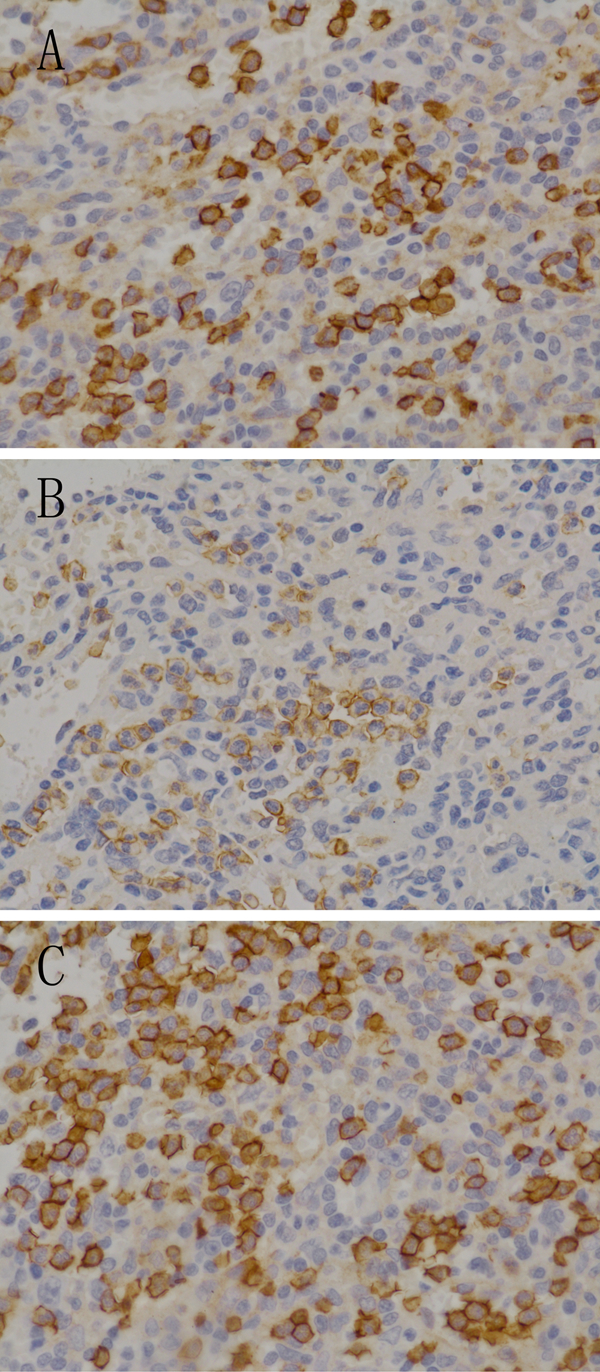

Supplement: Additional file 6 — Figure S6. Immmunohistochemical staining of CD88 in spleen. (A) Immmunohistochemical staining of CD88 in spleen by Abcam antibody (catalog number, ab11867). (B) Immmunohistochemical staining of CD88 in spleen by Abcam antibody (catalog number, ab11884). (C) Immmunohistochemical staining of CD88 in spleen by Santa Cruz antibody (catalog number, sc-70812). [file ar3873-S6.TIFF]

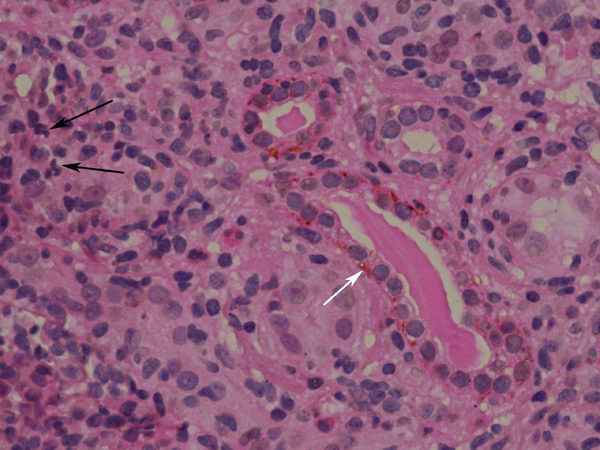

Supplement: Additional file 7 — Figure S7. Hematoxylin/eosin (HE) and CD88 counterstaining. CD88, open arrow; neutrophils, solid arrow. Magnification, ×400. [file ar3873-S7.TIFF]
